# P-1684. Perspectives from Sri Lankan physicians on current and future tools for diagnosing and managing lower respiratory tract infections

**DOI:** 10.1093/ofid/ofaf695.1858

**Published:** 2026-01-11

**Authors:** Dhammika R Palangasinghe, Warsha De Zoysa, U H Buddhika Y Dilshan, Jayani Gamage, Champica K Bodinayake, Maria D Iglesias-Ussel, Ajith Nagahawatte, Stefany Olague, Ruvini Kurukulasooriya, Senali Weerasinghe, James Samwel Ngocho, Hrishikesh Chakraborty, Armstrong Obale, Truls Ostbye, Susanna Naggie, Christopher W Woods, Evan Myers, L Gayani Tillekeratne, Melissa H Watt

**Affiliations:** Faculty of Medicine, University of Ruhuna, Galle, Southern Province, Sri Lanka; Faculty of Medicine, University of Ruhuna, Galle, Southern Province, Sri Lanka; Faculty of Medicine, University of Ruhuna, Galle, Southern Province, Sri Lanka; Faculty of Medicine, University of Ruhuna, Galle, Southern Province, Sri Lanka; Faculty of Medicine, University of Ruhuna, Galle, Southern Province, Sri Lanka; Duke University, Durham, North Carolina; University of Ruhuna, Galle, Southern Province, Sri Lanka; Duke Clinical Research Institute, Duke University, Durham, North Carolina; Faculty of Medicine, University of Ruhuna, Galle, Southern Province, Sri Lanka; Faculty of Medicine, University of Ruhuna, Galle, Southern Province, Sri Lanka; KCMC University, Moshi, Kilimanjaro, Tanzania; Duke University, Durham, North Carolina; Duke Global Health Institute, Durham, North Carolina; Duke University, Durham, North Carolina; DCRI/ School of Medicine, Durham, NC; Duke University, Durham, North Carolina; Duke University, Durham, North Carolina; Duke University, Durham, North Carolina; School of Medicine, University of Utah, Salt Lake City, Utah

## Abstract

**Background:**

Lower respiratory tract infections (LRTIs) impose a significant burden on healthcare systems, including in Sri Lanka. This study explored the attitudes, perceptions, and experiences of Sri Lankan physicians regarding their current approach to diagnosing and managing LRTIs (including the use of guidelines, rapid diagnostic tests [RDTs], and clinical decision support tools [CDSTs]). We also assessed physicians’ perspectives on the value and drawbacks of using electronic CDSTs (eCDSTs), with the goal of developing an eCDST in future to improve LRTI management.

**Methods:**

Semi-structured interviews were conducted with 15 physicians working in six public hospitals in the Western and Southern provinces of Sri Lanka. The interview guide topics included current decision-making processes in managing LRTIs, use of guidelines and pathogen-based & biomarker-based RDTs , experience with CDSTs, and the value and drawbacks of using eCDSTs in clinical practice.

**Results:**

Physicians reported relying mainly on clinical judgment when managing LRTIs and generally did not consult guidelines for typical cases. When used, guidelines served as a “framework” for decision-making and were often accessed online. Physicians felt that RDTs hold value, if cost is not a barrier. Twelve physicians reported using CDSTs, such as CURB-65, TIMI score, and Wells’ score, with most accessing them via MDCalc on mobile phones. Perceived benefits of eCDSTs included improved evidence-based decision-making, enhanced efficiency, and uniformity to standardize care and reduce bias. Potential drawbacks included the tools’ inability to account for patients' unique characteristics, local reliability concerns, inconsistent internet access, and unavailability of required inputs.

**Conclusion:**

Physicians relied on clinical judgment for LRTI management, using guidelines as a framework. RDTs were valued but limited by cost. CDSTs were commonly accessed and eCDSTs were seen as beneficial for evidence-based decision-making, efficiency, and standardization but raised concerns including applicability across differing patient characteristics, local relevance, internet access issues, and missing inputs. Effective eCDSTs for LRTI would need to address these limitations.

**Disclosures:**

Christopher W. Woods, MD, MPH, Biomeme: Patents on differentiating bacterial from viral infections|Biomeme: Ownership Interest|Biomeme: Stocks/Bonds (Private Company) Evan Myers, MD, MPH, Hologic, Inc: Advisor/Consultant|Merck, Inc: Advisor/Consultant|Moderna, Inc: Advisor/Consultant

